# Who's Leading This Dance?: Theorizing Automatic and Strategic Synchrony in Human-Exoskeleton Interactions

**DOI:** 10.3389/fpsyg.2021.624108

**Published:** 2021-02-17

**Authors:** Gavin Lawrence Kirkwood, Christopher D. Otmar, Mohemmad Hansia

**Affiliations:** Department of Communication, University of California, Santa Barbara, Santa Barbara, CA, United States

**Keywords:** synchrony, human-machine interaction, exoskeletons, emerging technologies, methodological challenges, human-robot interaction

## Abstract

Wearable robots are an emerging form of technology that allow organizations to combine the strength, precision, and performance of machines with the flexibility, intelligence, and problem-solving abilities of human wearers. Active exoskeletons are a type of wearable robot that gives wearers the ability to effortlessly lift up to 200 lbs., as well as perform other types of physically demanding tasks that would be too strenuous for most humans. Synchronization between exoskeleton suits and wearers is one of the most challenging requirements to operate these technologies effectively. In this conceptual paper, we extend interpersonal adaption theory (IAT) to the exoskeleton context and explicate (a) the antecedents that are most likely to shape synchrony in human-exoskeleton interactions, (b) automatic and strategic synchrony as adaptive behaviors in human-exoskeleton interactions, and (c) outcome variables that are especially important in these processes. Lastly, we offer a discussion of key methodological challenges for measuring synchrony in human-exoskeleton interactions and offer a future research agenda for this important area.

## Introduction

Synchrony has intrigued non-verbal communication researchers for several decades (Bernieri et al., 1988; Kendon, 1990; Bernieri and Rosenthal, 1991; Burgoon et al., 1995). According to interpersonal adaption theory (IAT), non-verbal synchrony is a type of reciprocal adaption that involves rhythmic patterns during an interaction where dyads coordinate their behaviors interdependently through matching, motor mimicry, and mirroring (Burgoon et al., 1995, 2014). Burgoon et al. (1995) explained that behavioral matching and motor mimicry are “in response to a stimulus and is often directed toward another person, mirroring is the imitation of another's body movements” (p. 26). In other words, synchrony involves two parties engaging in an interaction similarly as a result of the coordination in their behavioral patterns (Burgoon et al., 1995; Fujiwara et al., 2020). The main types of non-verbal synchrony include simultaneous behaviors between interactants, interaction rhythms that occur over the course of an interaction, and behavioral meshing that creates a meaningful whole [Bernieri et al., 1988; Bernieri and Rosenthal, 1991; see also the complementary scholarship on joint action in Knoblich et al. (2011)]. Past research on synchrony in human dyads has shown that non-verbal synchronous behaviors are used to signal interest, involvement, rapport, similarity, or approval (Kendon, 1970; Warner, 1992; Tickle-Degnen and Gavett, 2003), resulting in highly synchronous exchanges being mutually rewarding experiences for the interactants.

Empirical research investigating synchrony as a predictor of rapport, similarity, and approval in interpersonal interactions has also been leveraged in the field of human-robot interaction (HRI) (Kendon, 1970; Warner, 1992; Tickle-Degnen and Gavett, 2003; Hasnain et al., 2013; Bartneck et al., 2020b). Recent developments in robotics include making human-robot interactions reflect synchronous interactions between human dyads (Prepin and Revel, 2007; Hasnain et al., 2013). For example, the adaptable robotics for interaction analysis (ADRIANA) platform enables a robot to detect movements in human users and synchronize its movements automatically in real-time. The field of HRI has largely adopted the assumption that when robots automatically synchronize their movement to users, users will feel that interactions with these technologies are more natural and similar to human interactions (Hasnain et al., 2013).

Wearable robots are an emergent technology that has the potential to reshape relationships between humans and robots through the process of synchrony (de Looze et al., 2016). Although some organizations might prefer to develop autonomous robots that replace humans, exoskeletons offer the opportunity to combine the strength, precision, and performance of machines with the intelligence, agility, and creativity of a human workforce. Exoskeletons are defined as, “a wearable, external mechanical structure that enhances the power of a person” (de Looze et al., 2016, p. 671). According to Zaroug et al. (2019) exoskeletons are an emerging form of wearable robots in which synchrony challenges are especially crucial and salient for functionality.

There are many different types of designs for exoskeletons (e.g., tailored for lower limbs, full body suits) that can be passive or active. Passive exoskeletons do not have a power source, instead these devices rely on counterweights to collect energy from the wearer's own movements. This design is primarily used to support healthy postures or prevent injuries in repetitive work tasks (de Looze et al., 2016). In contrast to passive exoskeletons, active exoskeletons have a power source that can be used to dramatically augment human abilities or performance in physical tasks (Zaroug et al., 2019). Active exoskeletons were originally developed for military use including Raytheon's XOS 2 powered armored suit which provided protection, enhanced lifting power, and improved moving capabilities for wearers (Kopp, 2011). However, recent trends in active exoskeleton development are geared toward developing suits for industries that place heavy physical demands on workers and have high risk of injury (e.g., automobile manufacturing plants, distribution warehouses). A full-body active exoskeleton currently being developed for industry is the Guardian XO suit by Sarcos Corp (2019). The Guardian XO allows humans to easily lift up to 200 lbs. and offers features that allow the wearer to perform highly precise tasks with heavy tools or industry-specific equipment.

Synchrony can be difficult to achieve in human-exoskeleton interactions because it requires that an active exoskeleton can accurately detect when a wearer initiates movement and understand what type of movement the wearer wants the suit to perform. The wearer also will experience challenges in synchronizing their movement with the suit including how long to wait for the exoskeleton to respond to the wearer's movements, knowing how to move when an exoskeleton is performing a task (such as lifting a heavy item), and knowing when it is appropriate to initiate new movements. For instance, if a wearer is trying to lift a 200-lb piece of equipment they will need to initiate movement with their arms and wait for the exoskeleton to respond and pick up the item. Although there is likely to be variation in lag times across different active exoskeleton devices, even short lag times still require a wearer to be mindful of how their body movements may disrupt suit functionality. In this paper we leverage active exoskeletons as an illustrative example for other forms of wearable technology because of the high-stakes nature of synchrony in this context. These stakes include safety concerns from giving exoskeleton wearers such dramatically high levels of physical strength as well as how UX may reshape traditional blue-collar industries in which these suits are adopted.

Aligning with the theme of this special issue, it is clear that non-verbal communication scholars can fill significant knowledge gaps in human-exoskeleton interactions; however, non-verbal theories will need to be extended in order to do this important work. This paper frames the adaptation patterns between exoskeleton and human as non-verbal synchrony—opposed to the broader term of coordination—because we are specifically interested in how a person's rhythms are set into motion by active exoskeletons (Condon and Ogston, 1966; Kendon, 1990; Burgoon et al., 1995). We leverage an IAT framework to theorize non-verbal human-exoskeleton synchrony because it grounds these interactions in a communicative lens and offers rich heuristic value for explicating (a) the antecedents that are most likely to shape synchrony in human-exoskeleton interactions, (b) automatic and strategic synchrony as adaptive processes in human-exoskeleton interactions, and (c) outcome variables that are especially important in these processes. Understanding these antecedents, processes, and outcomes are important for exploring both how to make experiences with active exoskeletons more satisfying to users as well as how to increase user efficacy in the workplace. Lastly, we offer a discussion of key methodological challenges for measuring synchrony in human-exoskeleton interactions and offer directions for future research.

## Interpersonal Adaption Theory Framework

IAT builds upon previous adaption and coordination theories—such as expectancy violations theory (EVT; Burgoon and Hale, 1988) and communication accommodation theory (CAT; Giles, 1973)—while also leveraging biological principles, cognitive arousal, and social norms to explain adaptive dyadic behavior during interactions (Burgoon et al., 1995, 2017). Within IAT, synchrony is conceptualized as an adaptation behavior in which two people coordinate their behaviors interdependently through mirroring, matching, or reciprocity (Burgoon et al., 2014). Early research in synchrony found that synchronous behavior can be used to signal interest, involvement, rapport, similarity, or approval (Kendon, 1970). According to Burgoon et al. (1995) synchrony typically involves automatic biological responses, but can also be used strategically as it can, “function to regulate interaction and facilitate speech processing as well as express relational and emotional states” (p. 25). This suggests that IAT may be useful to further understand the regulated interactions between humans and exoskeletons. IAT provides a useful lens to understand the coordination in human-exoskeleton interactions through non-verbal synchrony—especially given the important role that strategic synchrony plays in this adaptive dyadic behavior (Burgoon et al., 1995).

### IAT and Emerging Technologies

Coordination is a fundamental part of satisfying interactions. The degree of coordination often predicts a variety of positive social and biological outcomes for the beneficiary. As an umbrella term that represents a broad range of concepts, coordination can be conceptualized as either communicative (e.g., mutual influencing interactions) or non-communicative (e.g., crew rowing) based upon the behaviors of the interactants. We use the term *adaption* to refer to the coordination of behaviors that are non-random and patterned in timing and form (Bernieri and Rosenthal, 1991) using a communicative lens (Burgoon et al., 1995). Although interpersonal adaption is often studied in face-to-face (Burgoon et al., 1995) and computer-mediated encounters (Dunbar et al., 2014), there is a growing body of scholarship that urges social scientists to further engage with HRI (Van Erp and Toet, 2013; Bartneck et al., 2020a). Given that all interpersonal interactions have a degree of coordination (Gatewood and Rosenwein, 1981; Bernieri and Rosenthal, 1991; Chartrand and Bargh, 1999), it is essential to construct a framework to better explain why, how, and to what effect interacting with emerging technologies have for humans.

Emerging technologies are increasingly designed to adapt to user preferences, understand and predict human behavior, and create optimal conditions for human-machine collaboration through a variety of approaches including machine learning via neural networks (Burrell, 2016), therefore making a theoretical framework for adaptive behaviors in HRI urgent. Unlike technologies where synchrony is automatic—such as robots designed with the ADRIANA platform—humans have more agency to synchronize with technologies they wear and operate. We recognize that exoskeletons are not the only emerging technology where synchrony is relevant, but it is clear that exoskeletons are a context in which synchrony is especially important for user safety and suit functionality. The stakes are higher for synchrony in human-exoskeleton interactions compared to other emerging technologies due to the closer proximal distance between users and suits. Additionally, unlike other technologies, how exoskeletons are designed will impact whether synchrony occurs automatically or if wearers are able to choose how and why they synchronize with the suit. This makes exoskeletons a helpful context for distinctions between strategic and automatic synchrony processes. Therefore, exoskeletons provide a prime exemplar for scholars to expand and rethink what it means to synchronize during an interaction and why particular antecedents of the user will predict a variety of outcomes to increase UX, comfortability, and efficacy with the technology.

One central assumption of IAT is that actors perform reciprocal or compensatory behaviors in response to the behaviors of the other partner in the interaction. Individuals are typically compelled toward reciprocal adaptions—such as synchrony—due to biological pressures and social expectations (e.g., politeness norms). IAT offers a detailed review of the conditions in which interactants are likely to partake in these reciprocal or compensatory behaviors based upon three antecedents that the person enters an interaction with: requirements (R), expectations (E), and desires (D).

### Requirements, Expectations, Desires

*Requirements* (R) are the individual perceptions of what needs to happen at any point during the interaction. These are often based on biological factors such as proximity to the speaker in order to hear what they are saying. Interactants also have particular *expectations* (E) of the interactions. Expectations can be thought of as socially based anticipations of the encounter and the communicator (Floyd and Burgoon, 1999). For example, many individuals expect their partner will have a particular level of interpersonal skills and abide by the social norms according to the culture, relationship, or profession. However, expectations can also be based upon past experiences with the person or previous knowledge they may have about the interactant. Finally, individuals enter interactions with particular *desires* (D) about what the interaction should accomplish by its conclusion. For example, an employee may enter an interaction with their supervisor to resolve issues with how work tasks are distributed.

#### Interaction Position

Requirements (R), expectations (E), and desires (D) coalesce in an overall evaluation called the *interaction position* (IP). This position is a “valenced behavioral predisposition” for an individual's own interaction behavior or what is anticipated from an interactional partner (Burgoon et al., 1995, p. 271). In other words, the IP is the perception an individual has about the interaction—and this perception is comprised of R, E, and D. Although IAT states that R of an interaction take precedence over D and E, requirements are often satisfied during an interaction, bringing D and E to play a more prominent role in determining the IP (Floyd and Burgoon, 1999). For instance, if an exoskeleton suit-wearer's R is met (e.g., the suit fits their body), the suit aligns with their E (e.g., the suit helps them accomplish work tasks), and satisfies their D (e.g., the suit is comfortable to wear), then we can expect the suit-wearer to have a positively valanced IP.

The IP is then compared to the *actual* (A) communication performed by the person during the interaction, which will determine whether the following interaction is patterned by reciprocal or compensatory adaptions. According to Burgoon et al. (2017), if A is more desirable than the IP, a partner is more likely to reciprocate the behavior, however, if the actual behavior is less desirable than the IP, the partner is predicted to compensate. The compensatory and reciprocal predictions are primarily based upon the magnitude of the discrepancy between the IP and the actual communication of the partner (A). Although small deviances are often tolerated during interactions, large deviances can lead to further assessment of the discrepancies' valence. IAT argues that large discrepancies should move toward whichever adaption pattern is more positively valanced for the interaction. Therefore, when the A > IP partners should display reciprocal behaviors (e.g., synchrony) and when A < IP, receivers should respond with compensatory behaviors (e.g., dissynchrony).

### Non-verbal Synchrony as Adaptation

Although these behaviors are typically unconscious, there are cases when participants of an interaction will consciously try to synchronize their behaviors with their partner. According to Burgoon et al. (1995) synchrony involves automatic biological responses, but can also be used strategically as a conscious adaptive behavior. According to IAT, a positive valence (A > IP) of the interaction between the user and the exoskeleton will lead to a reciprocal adaption—including synchrony—and may play a critical role in increasing the wearer's trust, rapport, and comfortability with this technology. However, the relationship between an exoskeleton and the wearer is complex and synchronization with an exoskeleton places unique physiological and psychological demands upon on the human throughout the interaction (Knight and Baber, 2005). Seemingly antithetical to human synchrony, human-exoskeleton synchrony suffers from the lack of a *mutually* rewarding experience and is one-sided. However, without optimal synchrony between the wearer and the exoskeleton, the user may grow tired and frustrated of using the machine and ultimately resort to not using the technology—defeating the central purpose of the exoskeleton to aid with physically demanding tasks.

**Figure 1:**
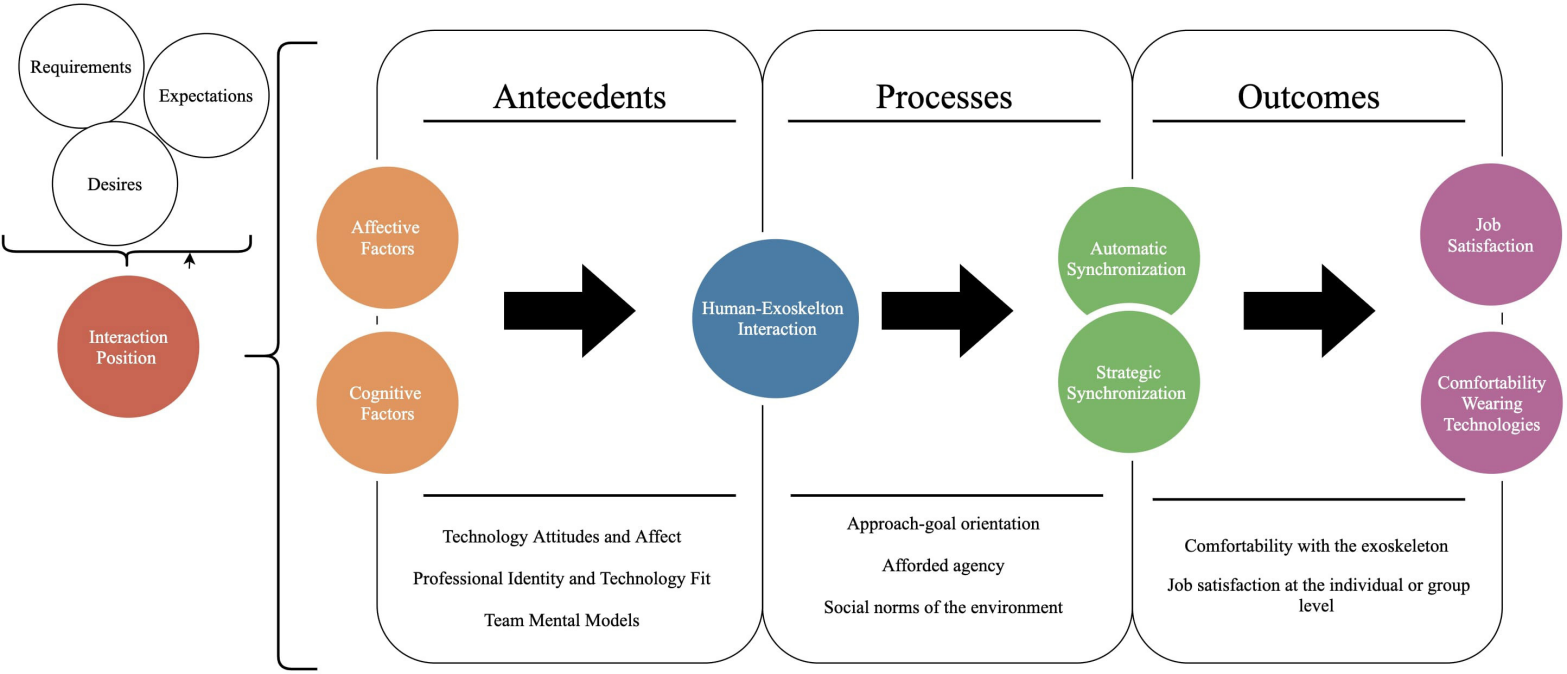
Chart detailing antecedents, processes, and outcomes that are likely to impact automatic and strategic synchronization in human-exoskeleton interaction.

The encounter between the user and exoskeleton is especially sensitive because of the close proximity between the wearer and the suit. Due to the exoskeleton often alleviating the pressures of physical labor (Upasani et al., 2019), the exoskeleton is close to the body and has the potential to violate an individual's space expectations. The skin is an especially important channel for social communication and “robot-initiated contact implies that the robot will enter the person's intimate space” (Chen et al., 2014, p. 141). Indeed, haptic contact with a machine in the workplace may have physical and psychological consequences (Upasani et al., 2019), such as claustrophobic feelings which could result in a rise in cortisol, skin irritation from adjusting the machine, or feeling stressed that the exoskeleton cannot be removed. Therefore, a priority researchers and practitioners alike should be to determine the antecedents, processes, and outcomes of the reciprocal adaption between exoskeleton and wearer in order to avoid the negative consequences of wearing an exoskeleton (see Figure 1). Important antecedents in these contexts include affective factors (e.g., feelings about or fears of new technologies) and cognitive factors (e.g., perceptions about wearable robotics). Processes include both automatic and strategic non-verbal synchronization behaviors between the human wearers and exoskeletons. Lastly, outcomes for synchrony in the exoskeleton context include comfortability with exoskeletons and overall job satisfaction.

## Antecedents in Human-Machine Synchrony

Unlike traditional forms of equipment used for heavy lifting in industrial or manufacturing environments (e.g., forklifts, dollies, or various carts), exoskeletons are a wearable technology (de Looze et al., 2016). Although traditional equipment (e.g., forklifts) often require an operator-tool dynamic with a clear physical distinction between operator and equipment, exoskeleton wearers have no physical distance between their bodies and the suit. The experience of wearing a technology involves multiple bodily sensations and can even make wearers feel as if the technology is an appendage of their own body (Smelik et al., 2016). For example, in a study exploring the affective impacts of wearable solar panels, Smelik et al. (2016) found that some participants reported the added spatial dimensions of the suit increased feelings of personal empowerment. According to Knight and Baber (2005), wearable technologies involve a myriad of cognitive and affective factors to consider for the safety and comfortability of wearer. Knight and Baber (2005) created a typology to operationalize the cognitive and affective factors for people who operate wearable computers including emotion, attachment, harm, perceived change, movement, and anxiety. Together, these six dimensions are likely to influence a wearer's ability to automatically or strategically synchronize with an exoskeleton (see Table 1 for specific exoskeleton applications for each dimension) but this is not an exhaustive list of relevant antecedents in this context. In addition to updating and extending Knight and Baber's (2005) typology to an exoskeleton context we also explicate other important affective and cognitive antecedents that are important in human-exoskeleton interactions.

**Table 1:** Applying Knight and Baber's (2005) comfort dimensions to the exoskeleton context.

**Comfort dimension**	**Wearer concerns**	**Exoskeleton application**
Emotion	“How do I feel when wearing the suit? “How do I feel when other people see me wearing the suit?”	The enhanced lifting capabilities may make wearers feel empowered. Wearers may feel insecure when wearing the suit in front of coworkers, especially if colleagues view the suit as unnecessary or as a crutch.
Attachment	“How often do I feel the suit moving on its own?” “When I initiate a movement, is there a lag time before the machine responds?”	Active exoskeletons use sensors to interpret the wearer's movements and act accordingly. Lag times between the wearer initiating a movement and the machine responding can feel constraining because the wearer would not want any additional movement to disrupt the process.
Harm	“Do I experience any pain or discomfort when wearing the suit?” “Do I have any disabilities or injuries that would cause discomfort in the suit?” “Does the suit accommodate my body type?”	Exoskeleton suits will range in their ability to accommodate all body types. Even if the exoskeleton is designed as “one-size fits all,” wearers who have sustained injuries or who have disabilities may be especially vulnerable to pain or discomfort.
Perceived Change	“Do I notice the suit moving while I'm wearing it?” “Is suit movement distracting or disorienting in any way?”	A feeling of disorientation may increase cognitive workload for wearers. If wearers are required to give increased focus while wearing the suit, it may compromise their ability to focus on work tasks. High levels of distraction or disorientation may mean that other coworkers or intelligent assistants will be required to help the wearer work effectively.
Movement	“Do I have free range of motion in the suit?” “Does the suit allow me to move in all the ways I need to work effectively?” “Does the suit restrict my ability to communicate with my coworkers?”	Exoskeleton suits are likely to vary in the range of motion they offer to wearers. To work effectively, wearers need to be able to move in ways that allow them to complete their work tasks. If range of motion is compromised, then a wearer's non-verbal communication might be compromised. Special consideration should be given in contexts where an exoskeleton reduces the social cues (e.g., gestures) required to communicate effectively.
Anxiety	“Does wearing the suit trigger any unique fears or anxieties?” “Am I afraid of or uncomfortable around new technologies?”	Wearing an exoskeleton may trigger unique anxieties or fears from individuals, such as claustrophobia. Other wearers may have anxieties and fears of the technology itself, which can be exacerbated when wearing the suit.

### Affective Antecedents

Synchrony during human-to-human interactions typically indicates positive affect, but we must first discuss the relationship between humans and exoskeletons. Emerging technologies regularly incite feelings of uncertainty and fear in humans (although this may stem from a lack of knowledge or motivation) and wearable robots such as exoskeletons are likely to be no exception. Fear of technology and robots are important to examine to gain a better understanding for how incorporating exoskeletons into a workplace can influence the humans involved. For the better part of a century, robots have been framed in fiction as intrusive and dangerous (Szollosy, 2015). According to Szollosy (2015), negative depictions of robots are presented more often to reflect human anxieties or uncertainties rather than any true technological developments. Because of these underlying anxieties that many individuals carry, wearable robot designers must consider how the technology will be received by the public— including what dominant expectations of the technology are held. In particular, robots often arouse strong emotions from people including fears of deskilling in the workplace, the loss of employment, or even larger existential threats to humanity (Szollosy, 2015).

Previous studies have investigated attitudes humans have toward robots. For instance, Nomura et al. (2008) conducted experiments where a human and a robot participated in basic interactions (i.e., meeting, self-disclosure, and physical contact). Negative attitudes and avoidance behavior exhibited from participants were measured. Negative attitudes influenced behavior toward robots as participants with negative attitudes spent significantly less time talking and touching the robot. In addition, gender differences were found as men who had negative attitudes about robots had higher instances of avoidance behaviors. On the other hand, the results also suggested that repeated interaction with robots can decrease avoidance behaviors over time. According to Nomura et al. (2008), these findings demonstrate that attitudes, perceptions, and other factors such as gender are important to consider in HRI research.

In addition to attitudes toward robotics that are influenced by larger cultural discourses or differences in gender, there are also affective dimensions that are unique to wearable technologies (Knight and Baber, 2005). Each of the dimensions that Knight and Baber (2005) offered have an affective impact on a wearer's experience with an active exoskeleton. The emotional dimension addresses the ways that wearable technology can make people feel when wearing the device and how they feel when others observe them wearing the suit. The attachment dimension addresses whether wearers feel the suit moving; an example would be if the suit or device moves autonomously or if users have control over suit movements. The harm dimension references any discomfort or pain that could arise as a result of the wearable device. The harm dimension includes high levels of individual variation as wearers who have sustained workplace injuries or disabilities are likely to experience this dimension differently than other wearers. The perceived change dimension encompasses any way that the wearable technology makes individuals feel different than how they would normally feel without the device (e.g., feelings of distraction, disorientation). The movement dimension addresses the ways in which a wearable technology can restrict the wearer's ability to move. Finally, the anxiety dimension addresses the remaining affective factors (e.g., claustrophobia) that could cause feelings of insecurity for the wearer. Taken together, these five dimensions, as well as larger cultural attitudes and perceptions of robotic technology, are likely to influence a wearer's emotional state when wearing an active exoskeleton.

### Cognitive Antecedents

#### Identity

One fundamental cognitive factor that is likely to shape synchrony between humans and exoskeletons in workplace settings is the wearer's sense of professional identity. According to Thoits and Virshup (1997) people develop meanings and expectations associated with work tasks based on how they understand their professional identities. These identities are shaped by social groups (in this case the groups at work) and the understanding of the self in relation to others (Tajfel and Turner, 1979). When it comes to interacting with technology, the technology's fit with a worker's identity tends to be just as important as a technology's fit to a task (Lin, 2012). According to Lin (2012) workers not only use technology that is necessary to complete a task but also use technologies that are consistent with how they view their work identities. It is important to recognize that social groups influence the task-technology and identity-technology fit: If a technology is perceived to not fit a certain identity, then using it to accomplish a task that goes against the expression of that identity would be deemed inappropriate (Fulk, 1993). For example, Upasani et al. (2019) found that agricultural workers were less likely to use exoskeleton technologies that, “do not seem to be work-related, and that are more ‘medical’ in their appearance” (p. 5). In other words, use of the technology violated the workers' understanding of their identities because they did not view a medical device as relevant to their work or role in the organization.

A wearer's sense of professional identity is likely to impact whether individuals synchronize with exoskeletons. When considering group membership and teamwork, identity often plays a large role in facilitating cooperation. CAT can be used to understand the relationship between intergroup dynamics and synchrony (Giles and Ogay, 2007). As a foundational theory of intergroup communication, CAT explains how people communicate and modify their communication toward different individuals. These modifications are made to converge or mimic the style of the other interactant, as well as the behaviors that meet the other interactants' perceived needs. Accommodation often occurs more when both participants in the interaction share a similar or compatible group identity.

According to Bernhold and Giles (2020), mimicry occurs with a goal for association as it overlaps with convergence and is synonymous with accommodation behaviors. Bernhold and Giles defined mimicry as the “unconscious imitation or mirroring of various nonverbal behaviors” (p. 62). If mimicry has the same results and goals as convergence, based on accommodation research, it can be suggested that identity would also influence synchrony in interactions. Particularly when discussing the success of a group, research on identity has illustrated that synchrony among individuals promotes prosociality. Prosociality can be defined as cooperation within individual dyads or between larger groups (Batson, 1998). Reddish et al. (2014) found that prosociality improved through synchrony among groups regardless of differences in group identity. The link between synchrony and accommodation has also been examined in a variety of contexts including parent communication with infants (baby talk) and communication between romantic partners (Locke, 1993; Lee et al., 2010). Other areas where these similarities have been researched include professional communication, and persuasion (Buller and Aune, 1988, 1992; Sparks, 1994). Accommodation and mimicry have been considered distinct but related concepts according to nonverbal research (Bernhold and Giles, 2020). Although research suggests that synchrony is likely more vital to group dynamics than perceived identity, it is still important to understand the identity affiliation or goal of the parties involved.

#### Team Mental Models

In addition to individual attitudes and perceptions toward a technology, group attitudes and perspectives will likely influence synchrony processes with exoskeletons in the workplace. According to Klimoski and Mohammed (1994) group members are related through shared cognition through team mental models (TMM). TMM is the idea that when working together, groups have conceptualizations and mental models that are either shared or compatible between group members. Research indicates that positive TMMs are positively associated with team coordination processes and overall performance (Mathieu et al., 2000; Fisher et al., 2012). As research suggests, if organizations can pinpoint general and contextual variables that can be linked to TMMs, and provide training and interventions to optimize TMMs, then one can anticipate highly coordinated and successful teams (Mathieu et al., 2000). During team compilation, team members interpret and obtain knowledge regarding their individual role, contextual social dynamics, what the task consists of, and what each team member brings to the group. This leads to an understanding of how they fit together and how they can accomplish tasks at hand. The higher degree of comprehension for these concepts, the more likely the TMM is positive. Positive TMM has been found to occur when team roles are understood early in team formation (Pearsall et al., 2010). If these same principles are addressed when an exoskeleton is designed and used, the results should be positive. The central distinction in the human-exoskeleton context would be that one member of the team is an active exoskeleton and would require the other members to be open to collaborating with it.

When the technology that is implemented is not introduced early and is seen as a threat to a team member's importance to the team, the results and perceptions may be negative. We suggest that mitigating potential tension between exoskeleton technology and team members includes early exposure, technical briefings, and plenty of hands-on experience. As with any technology, allowing time to first acclimate with the exoskeleton prior to implementation would give team members a better understanding of what the tech can and cannot accomplish. This then would give them an understanding on how to implement the tech into their TMM without feeling threatened that the suit will undermine the importance of human abilities or expertise in the group.

## Processes

Identifying the underlying processes of dyadic behavior has been a central aim for many nonverbal scholars that study coordination and adaption (e.g., Cappella, 1991; Arundale, 1996; Andersen, 1998). This makes sense considering that processual features are often thought of as the central component of human interaction [see Hewes (1979) for comments on process in social interaction research]. Identifying these features requires scrutiny of the simultaneous signaling as well as signal detection while the human and robot coordinate with one another. Patterson (2019) suggests that a key element to understanding the underlying process of HRI may be rooted in the goal compatibility between the human and robot. Complementary goals between humans and robots may increase the effectiveness and comfortability of the technology with the user. For the exoskeleton wearer, synchrony with the suit should be encouraged as the primary goal when wearing the device.

The seamless coordination of humans and technology could increase affect and trust in the machine, and therefore increase the likelihood that the wearer will be able to utilize and benefit from using an exoskeleton. Another factor that is unique to the exoskeleton-wearer interaction is the balance between the agency of the human and the abilities of the machine. If synchrony is to be the main goal during the coordination of the HRI, both the ability of the wearer to effectively control the robot and the capability of the exoskeleton to appropriately respond in kind to the wearer is key to the underlying process. It is not only important to understand the wearers' predispositions prior to using the exoskeleton and the consequences of the interaction, but critical to discuss how the synchronization unfolds throughout the course of the interaction. Burgoon et al. (1995) and Kellermann (1992) argue that adaption during interactions is mostly automatic but there is still some level of intent in every encounter. Therefore, the synchronization process can be both *automatic* and *strategic* representing both unconscious and conscious behaviors of exoskeleton wearers. Exoskeleton designers for private industry are still debating (a) what it means for humans to synchronize with exoskeletons, (b) how much control users should feel over an exoskeleton, and (c) how optimal synchrony can be achieved in these interactions. It remains to be seen which aspects of active exoskeletons are going to be designed to automatically synchronize with wearer movements or if these technologies will have some control over wearers in these interactions.

### Automatic Synchrony

Automaticity during an interaction are the features that are involuntary and unconscious by the agents (Kellermann, 1992). Therefore, automatic synchrony occurs without reflection from the wearer of the exoskeleton and they allocate very few cognitive resources to the behavior being enacted. These underlying biological mechanisms are not based upon social or cultural variations because they are more fundamental and rudimentary to the human communication process (Cappella, 1991). From the robotic side of the interaction, these are the automatic processes that the machine engages in to achieve its pre-determined goal. The importance of automatic synchrony in the HRI is noteworthy because it provides a sense of normalcy and, possibly, positive affect (such as feelings of confidence) throughout the exoskeleton interaction. Indeed, speech convergence tends to be associated with positive affect and the disruption of this convergence is often seen as jarring and abnormal (Feldstein et al., 1982). The more automatic the synchrony, the more engaged the user is expected to become while interacting with the exoskeleton. Additionally, the less time that the user needs in order to synchronize with the exoskeleton, the more cognitive resources freed up in order to focus on work tasks. In other words, the more automatic the synchrony between the wearer and the exoskeleton, the more the user will be able to concentrate on achieving the particular goal.

Cappella (1991) describes two different types of automated patterns during interactions: (1) stimulation regulation and (2) emotional responsiveness. Although originally proposed between two humans, the biological origins of these automatic patterns may still bring insight in what the exoskeleton wearer requires (R) expects (E), and desires (D) of the robot, therefore influencing the interactional position (IP) of the wearer. Stimulation regulation is the dyadic process in which a person controls the others' expressed level of activation. An example provided by Cappella (1991) is the tempo of the conversation—often measured by the rate of speech and the quickness of response. Bartneck et al. (2020a) argue contingency anthropomorphization of social robots can help users feel like the machine is partaking in appropriate regulation. For example, if the robot detects motion, “it should briefly look toward the origin of the movement” (p. 54). Similarly, the robot could be designed to improve stimulation regulation by using information about the user's previous motion patterns in order to tailor the exoskeleton experience. The stimulation regulation of the exoskeleton—or the responsiveness to the wearer throughout the time wearing the device—may be highly predictive of the user experience since it is likely that the user will desire (D) the robot to reflect the stimulation regulation expected (E) in dyadic human interaction.

The second automatic pattern is emotional responsiveness throughout the course of the interaction. Satisfying communication is often directly tied to partners successfully communicating felt emotions during an interaction (Andersen and Guerrero, 1998). Cappella (1991) describes this emotional responsiveness as the tendency to approach and withdraw from the emotional state of another. Although the wearer of the exoskeleton is the only part of the dyad that has biological origins, it is possible that the wearer of the exoskeleton is still expecting emotional responsiveness from the robot throughout the interaction. For example, if a user's body begins to stiffen because they are in pain while using the exoskeleton, it is critical that the exoskeleton is able to respond in kind to this new development, opposed to passively operating as if the emotional state of the wearer has not changed. Emotional responsiveness may also come from skin-conductance sensors or increase in heartbeat that indicate stress from the wearer (Bartneck et al., 2020a). Both stimulation regulation and emotional responsiveness as automatic processes can significantly influence the IP of the user and can increase the degree of synchrony depending on the actual behavior (A) of the device.

### Strategic Synchrony

Non-verbal synchrony can also be strategic and directed by the actor's goals. For example, a worker may be deliberate in their attempts to coordinate their movements with the exoskeleton in order to quickly finish a task and, in turn, increase productivity. Under these conditions, the wearer of the exoskeleton is intentional in their ability to adapt the machine and synchronize their movements in order to achieve a particular goal. Burgoon et al. (1995) explained that strategic synchrony regulates interactions and helps express relational and emotional states. If the wearer of the exoskeleton has positive affect toward the machine, the wearer may strategically attempt to synchronize movements with the robots in order to effectively accomplish the task that the exoskeleton and the wearer are jointly working on. The opposite may also be true. Increases in negative emotional states throughout an interaction have been associated with dissynchrony between adults and infants (Bernieri et al., 1988). It is possible that negative emotional states that unfold throughout an interaction between the exoskeleton and the wearer may cascade into an increasingly dyssynchronous encounter. However, there are underlying processes that take place as the interaction unfolds that motivate the wearer to strategically synchronize with the exoskeleton. We argue that three central motivations to strategically synchronize during the interaction with the exoskeleton are the (a) the levels of agency afforded to the exoskeleton wearer, (b) the goals of the exoskeleton wearer, and (c) social norms of the environment.

#### Agency

When we argue that agency is afforded to an exoskeleton user, we apply Gibson's (1986) theoretical concept of affordances to the exoskeleton context. Simply put, technological affordances refer to the intersection of what people believe they can accomplish with a technology and the technological features that either enable or constrain those goals. Treem and Leonardi (2013) explained that the technological affordance perspective is useful when exploring technology use because it “helps to explain why people using the same technology may engage in similar or disparate communication and work practices” (p. 146). In the exoskeleton context, the technical features in equipment design as well as the setting in which these technologies are adopted will likely impact the amount of agency afforded to human wearers.

According to Banks and de Graaf (2020), levels of agency afforded to humans and machines fundamentally impact human-machine collaboration. Agency typically refers to the ability of social actors that stem from resources, responsibilities, and capacity to reflect on situational context (Giddens, 1979). Within an exoskeleton context, it is important to recognize that strategic synchrony is the only form of synchrony that involves agency for human wearers. This means that in strategic synchrony contexts, workers have the ability to decide what ways they want to synchronize with the exoskeleton and how to enact those behaviors. In contrast, automatic synchrony means that regardless of what a wearer desires, synchrony will be achieved in the interaction. Although automatic synchrony gives lower levels of agency to the exoskeleton over the wearer, it could offer the seamless connection that wearers desire in the workplace or it may result in workers feeling disempowerment or a lack of control in their profession.

#### Goals

According to Lin et al. (2018), goals “shape people's behavior and direct their efforts toward different outcomes” (p. 314). Decades of research on interpersonal goals has shown that particular goal orientations often predict positive and negative affect toward interactions and relationships (Gable and Berkman, 2008). These orientations can also influence the interactants' non-verbal communication behaviors during an encounter, as well as an individual's understanding of the interaction once it has ended (Caughlin, 2010). Gable (2006) argued that two main goals drive most interpersonal interactions: approach goals and avoidance goals. Approach-goals tend to include positively valanced intentions and individuals seek to gain rewards from the interactions (e.g., affection). Avoidance-goals are characterized by evading threats during an interaction and are typically motivated by apprehension of conflict or failure. Individuals who are wearing exoskeletons that are more prone to avoidance-goals when interacting with robots could have a more negative experience. Further, they may be less likely to strategically synchronize their movements with the exoskeleton. However, wearers of exoskeletons who are more approach-goal oriented may be more likely to engage with the exoskeleton and strategically synchronize their movements to pursue the rewards of accomplishing the task. Apprehension to technology is likely a main predictor of an individuals' goal orientation throughout an interaction.

#### Norms

Social norms drive or constrain behavior and tend to be universally understood by particular members of a group (Horne, 2001). Emerging from interactions with other group members, social norm behaviors foster member expectations of themselves and others (Cialdini and Trost, 1998; Korte, 2010). Cialdini et al. (1990) even argued that these expectations, or “standards,” typically develop from observing others (i.e., descriptive norms). For example, treating robots as humanlike could be understood as a social norm since workers are more likely to perceive the machines they are working with as human if they see others doing the same (Bartneck et al., 2020a). The degree of motivation to strategically synchronize with an exoskeleton might depend upon the social norms of the environment in which the individual is situated. Therefore, if the social norm in the workplace is to strategically synchronize with exoskeletons, it is possible that the worker will be more inclined to follow suit.

#### Strategic Synchrony as Cooperation

Both the social norms of the environment and goals of exoskeleton wearers are primary motivators of strategic synchrony and can be explained by marrying the conditional cooperation norm and the reinforcement of cooperation model (Reddish et al., 2014). The conditional cooperation norm proposes that modern society functions from the underlying norm of cooperation. Simply stated, individuals are more likely to contribute if others in their environment are also contributing. Further, the higher the contribution rates observed by members, the more likely they will also contribute (Frey and Meier, 2004). The increase in contribution creates cooperation within a system, environment, or workplace. Similarly, from a goals-perspective, the reinforcement of cooperation model (Reddish et al., 2014) explains why spatial alignment amplifies cooperative responses from participants. Originally developed to understand shared intentionality during music and dance performances (Reddish et al., 2014), this model suggests that when there is a common goal to synchronize, the perception produces immediate feedback to the actor that cooperation is taking place. Increases in the feelings of joint rhythmic coordination reinforces successful cooperation and leads to participants feeling perceived similarity, trust, and confidence in their partners (Launay et al., 2016).

Of course, cooperation is typically a two-sided and mutually rewarding experience. However, extending IAT and strategic synchrony to human-exoskeleton interactions provides an opportunity to conceptualize cooperation from this new perspective. In contrast to human-human interactions, in the human-exoskeleton context perceived reciprocity from the exoskeleton may not influence the desire for cooperation unless users heavily anthropomorphize these technologies (Bartneck et al., 2020a). In this section we have conceptualized cooperation norms as the social norms that employees have toward exoskeletons at the team, group, or organizational level. If the social norm is to cooperate by strategically synchronizing with the exoskeleton, it is likely that this will motivate an individual to produce behaviors that foster a goal of cooperation between the wearer and the exoskeleton which will, in turn, reinforce the synchronization. This may be especially true if the individual is approach-goal oriented when wearing the exoskeleton. In sum, underlying strategic processes that unfold during the interaction to create synchrony can be explained by the individual goals and social norms of the environment in which the encounter takes place.

## Outcomes

When considering wearer synchrony and the use of active exoskeletons in the workplace, two main outcomes are especially salient. The first main outcome is wearer comfortability with the exoskeleton and the second main outcome is overall job satisfaction. Although there is currently no empirical research that explores the relationship between human-exoskeleton synchrony and these outcome variables, we offer some ideas on how automatic and strategic synchrony may influence these outcomes.

### Comfortability With Wearable Technologies

Knight and Baber (2005) explained that it can be difficult for designers to create wearable technologies that multiple stakeholder groups feel comfortable wearing. These challenges are partly attributed to individual variations between users, especially as we expect to see large variations among users in the antecedent variables we propose. When we refer to comfortability in the exoskeleton context, we recognize two main distinctions. One aspect of comfortability involves how comfortable wearers feel when wearing the exoskeleton and the second aspect refers to how comfortable users feel when using the exoskeleton in a work environment. When it comes to comfortability when using the exoskeleton, affective and physiological factors are especially important as uncomfortability or pain can lead to musculoskeletal disorders for wearers.

For comfortability when using the suit in the workplace, automatic synchrony or strategic synchrony likely will influence whether employees want to use the exoskeleton and how they want to use it. For instance, automatic synchrony processes may make individuals feel less burdened on a cognitive level which could help them pay closer attention to their surroundings and feel more comfortable using the exoskeleton around coworkers. However, the opposite may also be true. Automatic synchrony could may make wearers feel burdened to understand how the machine is functioning, resulting in apprehension toward using the suit in the workplace. Strategic synchrony could also have nuanced impacts on comfortability for using the exoskeleton in work tasks. On one hand, the ability to strategically synchronize with an exoskeleton may help users feel more empowered and in control of the suit's movements leading to feelings of comfortability with the suit around coworkers. However, strategic synchrony may also place too many cognitive demands on the wearer and decrease their ability to perceive their surroundings which could lead to apprehension when wearing the suit.

### Job Satisfaction

We expect that automatic and strategic synchrony could also have nuanced impacts on job satisfaction for wearers. Organizational research showcases that when employees feel a lack of control, agency or autonomy in the workplace, they are more likely to experience stress, burnout, and report decreased levels of overall of job satisfaction (Chen and Silverthorne, 2008; Mahon, 2014). If strategic synchrony can help increase feelings of autonomy and an employee's internal locus of control, then employees who can choose to engage in synchrony may be more satisfied in their work. But if strategic synchrony is too costly on a worker's physical and cognitive energy, they may not feel in control of the suit which could lead to stress and lower levels of overall job satisfaction. There are also nuanced possibilities on the impact of automatic synchrony and overall job satisfaction. For instance, if workers feel more in control of the tasks they complete with an exoskeleton in automatic synchronization conditions, then we expect overall job satisfaction to increase. However, if automatic synchrony compromises feelings of autonomy and control for wearers then overall job satisfaction is likely to decrease. Non-verbal scholars are well-positioned to examine the tradeoffs that employees make in automatic and strategic synchronization conditions and how these processes ultimately impact working conditions for wearers.

## Methodological Challenges

Exoskeletons are likely to dramatically shift how work is conducted in traditional blue-collar industries. Although there has yet to be research on synchrony between humans and wearable technologies—including exoskeletons—there are two key methodological challenges that non-verbal researchers will face in this area. The two key challenges are determining the relationship between perceptions of synchrony and actual instances of synchrony, and the technical challenge of separating human and machine datapoints for analysis.

### Perceptions in Synchrony

One key challenge of synchrony research in HRI contexts is to understand whether the perceptions of synchrony that wearers report actually match levels of synchrony with exoskeleton. Although perceptions of synchrony are the least expensive and easiest data to obtain, it may not provide the complete picture of how synchrony functions in HRI. However, automated wavelet spectrum analysis in non-verbal communication research could be a useful tool for overcoming this specific challenge (Fujiwara and Daibo, 2016). Fujiwara and Daibo (2016) explained that early research on synchrony involved human coders observing interactions and coding for synchrony behaviors which was ultimately time-intensive and costly. In contrast to these traditional methods, researchers that use wavelet spectrum analysis leverage in-depth sensors and can automate the coding of synchrony behaviors. When conducting research on synchrony in wearable technologies, human coders would not be able to distinguish movements of the human wearer from the exoskeleton suit because of close proxemics, so an automated method such as wavelet spectrum analysis will likely be needed to conduct this research.

### Separating Datapoints

The second main methodological challenge involves the ability to separate datapoints from the wearer's and suit's movements and collect both types of data for analysis. Active exoskeletons are already being designed with many sophisticated sensors that detect, anticipate, and react to wearer movements so the main challenge would be to log wearer movements independently of exoskeleton movements. One way this could be possible is to have sensors on the wearer that are independent from sensors (such as sensors on the clothing a wearer has underneath the exoskeleton suit) on the exoskeleton so that both types of data could be analyzed as separate units. The separation of these datapoints would be crucial for researchers to be able to use automated synchrony methods that do not require manual human coding [see Fujiwara and Daibo (2016) for an example of automated wavelet spectrum analysis in synchrony research]. Overcoming these two methodological challenges are crucial for conducting synchrony research in human-exoskeleton interactions.

## Future Research Agenda

If research on synchronization in human-machine dyads is in its infancy, then research on synchronization in human-exoskeleton dyads is still in conception. We encourage non-verbal communication researchers to critically engage with wearable technologies and explore how traditional non-verbal communication theories can be extended to new contexts. We propose two key areas of future research that will help shape knowledge of synchrony between humans and exoskeletons including testing and adding complexity to the IAT framework we propose.

### Testing the IAT Framework

We encourage non-verbal communication scholars who are interested in human-exoskeleton interactions to empirically test the antecedents, processes, and outcomes we offer in this piece. In designing this program of research, testing the interaction position including the requirements, expectations, and desires that users have before interacting with an exoskeleton would be a critical first step to testing this overall framework.

#### Antecedents

Next, we encourage researchers to explore the antecedent variables that are likely to have the biggest impact on synchrony in human-exoskeleton interactions. In the area of affective antecedents, prior HRI research has shown that humans may be predisposed with negative attitudes, anxieties, or negative affect toward robotic technologies (Nomura et al., 2008; Bartneck et al., 2020a). Scales such as the negative attitudes toward robots scale (NARS) or the robot anxiety scale (RAS) have been tested and used as ways to gauge attitudes and anxieties toward robots (Nomura et al., 2008). Although they can give insight for exoskeleton technology, these measures are still specific to fully automated robots. More research much be done to understand whether these attitudes, anxieties, and affect toward autonomous robots are transferable to wearable technologies more generally and specifically exoskeletons. A logical step in exoskeleton research would be to determine differences that people may have when discussing negative attitudes and anxieties toward robots and exoskeletons. If they shown to be similar, then many of the implications the scales have for HRI could be extended to exoskeleton research. Once the scales are also proven to be valid indicators of attitudes and anxieties toward exoskeletons, a potential program of research can be conducted to further determine how different attributes of humans influence how they score on these scales. The transferability of these concepts is especially important to explore considering the complex physiological, affective, and cognitive factors in wearable technologies (Knight and Baber, 2005).

Also, worth addressing is Asher's et al. (2020) research on how to lower levels of anxiety in HRI. Through an analysis of videos, Asher et al. explored how individuals with social anxiety did not improve non-verbal synchrony when having closeness-generating conversations but did improve when having small-talk conversations. This line of inquiry could potentially give insight on how to design interventions that can lower anxiety between humans and wearable robots as well as increase positive attitudes or affect toward these technologies. Isolating the relationship between these interventions and synchrony will be crucial for understanding how organizations should orient employees toward exoskeletons as well as how trainings can help users improve their ability to synchronize with the technology.

In regard to cognitive antecedents, we urge researchers to explore how organizations can help teams develop more positive TMMs during active exoskeleton adoption (Mathieu et al., 2000). This area of research must involve understanding how beliefs about individual roles, perceptions of tasks, and collaboration norms within traditional blue-collar work changes with active exoskeleton adoption at the team, department, or organizational level. Researchers interested in these issues should also consider when is the proper time to introduce interventions designed to increase the positivity of TMMs in this context. Although we know from Pearsall et al. (2010) that positive TMM is more likely to occur when the understanding of roles occurs early in the team's history, we do not know how early these interventions should be introduced to be most effective. For instance, in the active exoskeleton context, it is unclear when employees should be exposed to the technology before it is integrated in work practices as well as how much time teams should generally have to test the technology without worrying about hitting performance metrics typically required in their work.

#### Processes

Exploring the relationships between agency in automatic and strategic synchrony is also potentially a fruitful program of research. Although past organizational research showcases examples where employees need appropriate levels of agency and autonomy to enjoy their work, we simply do not have enough information to understand whether this is transferable to the use of active exoskeletons in the workplace (Chen and Silverthorne, 2008; Mahon, 2014). Research in this area should specifically explore whether users of wearable technology feel more agency in their roles if the technology automatically synchronizes with their movements or if workers feel more empowered when they can strategically synchronize with the technology. This line of inquiry is especially complex because it questions the relationship between active exoskeleton use and professional identity. Another complex dimension in this program of research is the tradeoff that employees make between receiving enhanced physical capabilities when synchronizing with active exoskeletons and the costs of cognitive energy that wearers experience when using exoskeletons to complete tasks.

#### Outcomes

Lastly, when designing a program of research to test the IAT framework it is important that researchers explore how different types (automatic or strategic) and levels of synchrony between humans and exoskeletons impact the outcome variables we suggest. For the outcome of comfortability for exoskeletons wearers, we have extended Knight and Baber's (2005) typology of comfortability dimensions for wearable technologies to the active exoskeleton context and have provided concrete examples of how these dimensions could be relevant in these interactions (See Table 1). We suggest that this line of inquiry first be conducted from an inductive or exploratory approach as there may be some important comfort dimensions relevant to the human-exoskeleton context that have not been mentioned in prior scholarship or research.

When designing a program of research addressing levels of job satisfaction, we know from prior organizational research that when employees feel a lack of control, agency or autonomy in the workplace, they are more likely to experience stress, burnout, and report decreased levels of overall of job satisfaction (Chen and Silverthorne, 2008; Mahon, 2014); but we do not the extent to which automatic or strategic types of synchrony impact levels of agency, autonomy, and control that workers perceive and experience. Special consideration should also be paid to variations in professional identity and industry affiliation play in predicting the relationship between type of synchrony (whether automatic or strategic) and agency, autonomy, and control in the workplace.

We expect that individuals who are used to high levels of agency, autonomy, and control (such as trainers, supervisors, or managers) may be more sensitive to changes in these variables and can be more susceptible to changes in job satisfaction when active exoskeletons are adopted. This may be partially attributed to the ways that active exoskeleton adoption can disrupt expertise in organizations. Past research on robotics have shown that when robots take larger roles in complex tasks, expertise in organizations can be disrupted in both positive and negative ways (Davenport, 2018; Beane, 2019). In an ethnographic case study on a cadre of initiate surgeons, Beane (2019) found that the new collaborative relationships with robots in surgery interrupted the normal training process for surgeons and required that they pre-maturely chose specific expertise. Due to the emerging nature of active exoskeleton technology in blue-collar industries researchers should explore (a) how disruptive these technologies will be and (b) how the disruptive nature will impact job satisfaction in these environments across different types and groups of employees.

### Adding Complexity to the IAT Framework

The main challenge of theorizing about a cutting-edge technology such as active exoskeletons is that the complexity of frameworks that can be introduced in these contexts has limitations. Although we hope that the robust IAT framework we apply to the human-exoskeleton context helps inspire new and provocative types of research, we recognize that our framework is not an all-exhaustive list of the important concepts and variables for this context. We briefly mention two concepts that could be introduced to add more complexity to the IAT framework we propose.

#### Entrainment

Entrainment is defined as, “a process that leads to temporal coordination of two actors' behavior, in particular, synchronization, even in the absence of a direct mechanical coupling.” (Knoblich et al., 2011, p. 63). It is important for researchers to consider how entrainment and temporal dimensions vary across different types of powered exoskeletons and the goals or motivations of the wearer. For instance, some types of powered exoskeletons are designed for medical rehabilitation for wearers who have sustained serious injuries. For instance, the Indego personal suit enables individuals with spinal cord injuries to stand and walk independently (Parker Hannifin Corp, 2019). Active exoskeletons designed to help injured individuals walk are very different than active exoskeletons designed to give workers super-human levels of strength in the workplace. It is clear that differences in the design of these medical active exoskeletons and motivations of wearers will impact the process of entrainment, the temporal nature of tasks that the exoskeleton is used for, and the type of synchrony (automatic or strategic) that is available in this context.

#### Process Interactions

Given that designers of active exoskeletons for private industry are still debating how responsive and how much control these technologies should have during interactions, we have conceptualized automatic and strategic synchrony as two distinct processes. However, it is possible that wearers may find that certain parts of an exoskeleton automatically synchronize with their movements more than others, or that performing some types of tasks give them more control over the exoskeleton. We recommend that researchers who are interested in synchrony in the active exoskeleton context should be open to considering how both types of synchrony may be present in the same piece of technology as well as how the interactions between these processes influences the antecedents and outcomes we mention.

## Conclusion

Emerging technologies are becoming increasingly complex, not only in how the technology operates but also in how these technologies make people *feel*. Advancements in emerging technologies such as active exoskeletons illustrate that collaborative relationships between humans and machines are likely to become more important across a variety of professional settings. We have applied IAT to human-exoskeleton interactions in order to offer an in-depth and illustrative example for how traditional non-verbal communication theories can be reimagined in new technological contexts, but we certainly do not think these efforts should only be scoped to exoskeleton technologies. Certainly, there are a multitude of technological contexts, non-verbal communication variables, and methodological challenges that should be considered by non-verbal communication researchers and practitioners alike. We hope that the initial insights we have provided help inspire researchers to keep interrogating non-verbal communication theories in a rapidly changing world and continue to ensure our field has the relevance needed to meet the challenges of the future.

## Data Availability Statement

The original contributions presented in the study are included in the article/supplementary material, further inquiries can be directed to the corresponding author/s.

## Author Contributions

GK leveraged expertise with emerging technologies to conceptualize the central argument for this piece, including how to extend IAT to the human-exoskeleton context. CO leveraged expertise in synchrony research to explicate key concepts and variables that were foundational in our theoretical argument and designed figures to help clarify theoretical arguments. MH leveraged expertise in nonverbal communication to identify important antecedents in human-exoskeleton interactions. All authors contributed to the article and approved the submitted version.

## Conflict of Interest

The authors declare that the research was conducted in the absence of any commercial or financial relationships that could be construed as a potential conflict of interest.
